# Two Novel Variants in *YARS2* Gene Are Responsible for an Extended MLASA Phenotype with Pancreatic Insufficiency

**DOI:** 10.3390/jcm10163471

**Published:** 2021-08-05

**Authors:** Lidia Carreño-Gago, Diana Luz Juárez-Flores, Josep Maria Grau, Javier Ramón, Ester Lozano, Ferran Vila-Julià, Ramon Martí, Glòria Garrabou, Elena Garcia-Arumí

**Affiliations:** 1 Research Group on Neuromuscular and Mitochondrial Disorders, Vall d’Hebron Institut de Recerca (VHIR), Vall d’Hebron Hospital Universitari, Universitat Autònoma de Barcelona, 08035 Barcelona, Spain; lidiacarre27@gmail.com (L.C.-G.); javier.ramon@vhir.org (J.R.); ferran.vila@vhir.org (F.V.-J.); ramon.marti@vhir.org (R.M.); 2 Centro de Investigación Biomédica en Red de Enfermedades Raras (CIBERER), Instituto de Salud Carlos III, 08036 Barcelona, Spain; dian.juarez@icloud.com (D.L.J.-F.); garrabou@clinic.cat (G.G.); 3 Muscle Research and Mitochondrial Function Laboratory, Cellex-IDIBAPS, Faculty of Medicine and Health Science-University of Barcelona, Internal Medicine Service-Hospital Clínic of Barcelona, 08036 Barcelona, Spain; 4 Department of Cell Biology, Physiology and Immunology, University of Barcelona, 08028 Barcelona, Spain; elozano@ub.edu; 5 Department of Clinical and Molecular Genetics, Vall d’Hebron Hospital Universitari, 08035 Barcelona, Spain

**Keywords:** mitochondrial aminocyl-tRNA synthetase, novel pathogenic variant, Pearson’s syndrome

## Abstract

Pathogenic variants in the mitochondrial tyrosyl-tRNA synthetase gene (*YARS2*) were associated with myopathy, lactic acidosis, and sideroblastic anemia (MLASA). However, patients can present mitochondrial myopathy, with exercise intolerance and muscle weakness, leading from mild to lethal phenotypes. Genes implicated in mtDNA replication were studied by Next Generation Sequencing (NGS) and whole exome sequence with the TruSeq Rapid Exome kit (Illumina, San Diego, CA, USA). Mitochondrial protein translation was studied following the Sasarman and Shoubridge protocol and oxygen consumption rates with Agilent Seahorse XF24 Analyzer Mitostress Test, (Agilent, Santa Clara, CA, USA). We report two siblings with two novel compound heterozygous pathogenic variants in *YARS2* gene: a single nucleotide deletion in exon 1, c.314delG (p.(Gly105Alafs*4)), which creates a premature stop codon in the amino acid 109, and a single nucleotide change in exon 5 c.1391T>C (p.(Ile464Thr)), that cause a missense variant in amino acid 464. We demonstrate the pathogenicity of these new variants associated with reduced *YARS2* mRNA transcript, reduced mitochondrial protein translation and dysfunctional organelle function. These pathogenic variants are responsible for late onset MLASA, herein accompanied by pancreatic insufficiency, observed in both brothers, clinically considered as Pearson’s syndrome. Molecular study of *YARS2* gene should be considered in patients presenting Pearson’s syndrome characteristics and MLASA related phenotypes.

## 1. Introduction

Mitochondria are ubiquitous organelles that integrate various metabolic pathways (oxidative phosphorylation (OXPHOS), fatty acid oxidation, Krebs cycle, urea cycle, gluconeogenesis and ketogenesis) [1] and are involved in different crucial functions for cells (thermogenesis, biosynthesis of heme and iron–sulfur clusters (Fe–S), calcium homeostasis and apoptosis) [2].

A particularity of these organelles is that mitochondrial protein synthesis and, thus, mitochondrial function, is controlled by two different genomes: mitochondrial DNA (mtDNA) and nuclear DNA (nDNA). Mitochondrial DNA consists of 37 genes, of which only 13 codify subunits of the OXPHOS system while 2 rRNA and 22 tRNA are coded for their translation [3]. The other mitochondrial proteins are codified by nDNA, synthesized in the cytosol and imported into the mitochondrion.

To maintain an adequate pool of mitochondrial proteins, mtDNA replication must be continuous and highly effective. One of the key group proteins involved in mitochondrial translation are the mitochondrial aminocyl-tRNA synthetases (aaRSs) [4]. Mitochondrial aaRSs catalyze the attachment of the amino acids to their cognate tRNA in a reaction known as aminoacylation of the tRNAs. Nuclear genes codify for all mitochondrial aaRSs. Pathogenic variants in genes coding for mitochondrial aaRSs have recently been shown to account for an increasing number of mitochondrial diseases, with phenotypic heterogeneity and significant tissue specificity [5].

Inherited sideroblastic anemia has been associated with pathogenic variants in several genes such as *ABCB7*, *ALAS2*, *GLRX5*, *YARS2*, *PUS1*, *SLC25A38*, *TRNT1* and *SLC19A2* [6], but the phenotype is variable with overlapping clinical presentations. MLASA syndrome (myopathy, lactic acidosis and sideroblastic anemia) was first described as associated with *PUS1* [7] pathogenic variants, and later in patients with similar phenotype *YARS2* pathogenic variants [8]. Moreover, heteroplasmic m.8969G>A variant in *MT-ATP6* gene and *LARS2* pathogenic variants were found in MLASA patients [9,10].

*YARS2* encodes the mitochondrial tyrosyl-tRNA synthetase protein, which is involved in the tyrosine binding to its analogous tRNA in the mitochondrion. It is encoded in chromosome 12p11.21 and pathogenic variants in this gene (OMIM#610957) have been associated with disease in a recessive mode of inherence (homozygous or compound heterozygous). Pathogenic variants have been described as previously associated with infantile- to childhood-onset autosomal recessive MLASA2 syndrome (OMIM#613561) [8,11] with mitochondrial respiratory chain (MRC) deficiency [12]. Penetrance for the most common pathogenic variant is complete, but a wide phenotypic variability has been evidenced. Within the variable phenotype, cardiomyopathy, diarrhea, hepatosplenomegaly and ovarian failure have been described (HGMD database) [8,11,12,13,14,15,16,17]. It has been hypothesized that mitochondrial mtDNA haplotype background may influence phenotypic expression, and haplotype H was associated with slower progression and less severe phenotype in YARS2 patients [12,13], but further evidence is needed to confirm this association.

In this study, we present a family with two affected brothers (P1 and P2) with late-onset MLASA and pancreatic insufficiency, previously clinically diagnosed as Pearson’s syndrome. Multiple mtDNA deletions were previously reported in these patients and their unaffected mother (P3), accompanied by multiple enzymatic respiratory chain deficiencies in the case of both siblings [18]. Current examination by whole exome sequencing (WES) revealed that P1 and P2 were compound heterozygous for *YARS2* potential pathogenic variants.

## 2. Materials and Methods

This study was carried out by the tenets of the Declaration of Helsinki and was approved by the local bioethics committee of our hospital. Informed consent was obtained from the patients, and age-matched controls. Exclusion criteria of age-matched controls were the presence of any concomitant disease, contact with mitochondrial toxics and family history of mitochondrial disease or drug abuse.

### 2.1. Source of Samples

Blood sample and fibroblasts were obtained from P1. Muscle biopsy was performed in P2 following standardized procedures and was immediately cryopreserved at −80 °C. Mouth swab was taken from P3.

Fibroblasts were obtained from P1 and matched controls by a skin punch biopsy, as previously reported [19,20] and were grown in 25 mM glucose DMEM medium (Gibco, Life Technologies, Waltham, MA, USA) supplemented with 10% heat-inactivated fetal bovine serum and 1% penicillin-streptomycin at 37 °C, in a humidified 5% CO_2_ air incubator, until 80% optimal confluence was reached.

### 2.2. DNA and RNA Isolation

Total DNA was obtained by standard phenol chloroform procedure from healthy controls and patients’ samples; blood and fibroblasts (P1), muscle biopsy (P2) and mouth swab (P3). Total RNA was isolated from P1 and control fibroblasts with RNAeasy mini kit (Qiagen, Venlo, The Netherlands) and cDNA was obtained by retro-transcription performed with High-capacity cDNA Reverse transcription kit (Applied Biosystems, Foster City, CA, USA), as previously reported [19].

### 2.3. mtDNA Maintenance Genes Custom Panel

Exonic and the flanking intronic regions of 17 genes (*DGUOK*, *DNA2*, *FBXL4*, *MGME1*, *MNF2*, *MPV17, OPA1*, *POLG, POLG2*, *RNASEH1*, *RRM2B*, *SLC25A4*, *SPG7*, *SUCLA2*, *SUCLG1*, *TK2* and *TWNK*) implicated in mtDNA replication and maintenance were studied by Next Generation Sequencing, using a previous designed panel with GeneRead Custom Panel V2 (Qiagen) technology. Briefly, 20 ng of total DNA were amplified by the panel. The resultant amplicons were purified and used to prepare individual libraries with NEBNext Ultra II DNA for Illumina Library Prep Kit (New England Biolabs, Ipswich, MA, USA). Independent libraries were quantified with Qubit^®^ dsDNA HS Assay Kit (Life Technologies), normalized to 4 nM concentration and pooled. The pooled libraries were sequenced into MiSeq platform (Illumina, San Diego, CA, USA). Data analyses were performed with GeneRead Targeted Enrichment Exon Panel Data Analysis (Qiagen, Venlo, NL, USA) software and results were annotated with a laboratory pipeline.

### 2.4. Whole Mitochondrial mtDNA Sequencing

In patient’s muscle DNA (P2), whole mitochondrial genome was amplified in a single amplicon by long- range PCR using the Takara LA PCR kit as previously described [20]. 10–20 ng of DNA is needed per reaction. Qubit 2.0 Fluorometer (Thermo Fisher Scientific, Waltham, MA, USA) was used for quantifying the amplicons and normalized each sample to 0.2 ng/µL. Amplicons were used for sample library preparation with Nextera XT DNA Sample Preparation kit (Illumina, San Diego, CA, USA), following the manufacturer’s instructions. 1 ng of amplified mtDNA is needed to prepare each library consisting of fragments of 150 pb. PCR amplicons were cleaned up with Ampure beads XT, and the libraries were normalized and pooled. The pooled libraries were loaded into the MiSeq Reagent kit V2 (300 cycles and 2 × 150 chemistries) (Illumina, San Diego, CA, USA) and sequencing proceeded in the MiSeq platform (Illumina, San Diego, CA, USA). In general, 40 libraries were multiplexed to obtain 5000X medium coverage. On-board software converted raw data to BAM/BAI and VCF files using GATK tools. These files were analysed by MiSeq Reporter Software.

### 2.5. Whole Exome Sequence

Whole exome was studied with TruSeq methodology in patient’s muscle DNA (P2). The exonic and the flanking intronic regions were captured and the DNA libraries were prepared with the TruSeq Rapid Exome kit (Illumina, San Diego, CA, USA), following manufacturer’s specifications. The sequencing was carried out in a NextSeq platform, data analyses were performed with DNANexus platform and we used a laboratory pipeline to variant annotation. The candidate pathogenic variants found were confirmed by Sanger sequencing. The two candidate variants found in *YARS2* gene were also analysed by Sanger sequencing in mother buccal mucosal DNA (P3) and in blood and fibroblast DNA and cDNA extracted from P1.

### 2.6. Expression of YARS2 mRNA by Real Time PCR (qPCR)

Quantification of *YARS2* gene expression was performed with the resultant fibroblast cDNA by qPCR in an ABI Prism 7900HT Sequence Detection System, with TaqMan Universal Master Mix, in a 12.5 µL reaction, using two different TaqMan *YARS2* gene expression assays (Hs01126899_m1 and Hs01126901_m1, Thermo Fisher). PPIA assay (Hs99999904_m1, Thermo Fisher Scientific) was performed to normalize the results and used as endogenous control. Relative gene expression quantification was calculated with Ct data by RQ Manager 1.2.1 Software (Thermo Fisher Scientific). Every analysis was performed in quadruplicate.

### 2.7. Mitochondrial Translation

Mitochondrial protein translation was studied in fibroblasts (P1 and 4 controls) three times, following the Sasarman and Shoubridge protocol [21]. We seeded a 60 mm culture plate per cell line of fibroblasts until they reached 80–90% confluence. Cells were washed twice with PBS 1X and incubated for 30 min with 2 mL of labelling media (DMEM without methionine and cysteine, 1X GlutaMax, 110 mg/L sodium pyruvate), equilibrated previously for 30 min to 5% CO_2_ and 37 °C. One hundred µL of 2 mg/mL emetine was added and incubated for 5 min followed by 60 min of incubation with 400 µCi of EasyTag labelling mixture. Next, the media was removed and replaced by 5 mL of equilibrated DMEM High glucose media with 10% FBS for 10 min. Cells were washed 3 times with PBS and, using a cell lifter, cells were collected by 750 µL to cold PBS 1X. Cells were centrifuged at 1500× *g* for 10 min at 4 °C, the supernatant was removed, and the pellet was resuspended in 100 µL cold PBS. We determined the protein concentration by BCA Protein Assay Kit and the volume corresponding at 100 µg of protein was centrifuged at 20,000× *g* for 20 min at 4 °C. The resultant pellet was resuspended in 50 µL of loading buffer (Tris 100 mM pH 6.8, SDS 4%, 20% glycerol, DTT 1M, 1% bromophenol blue) and sonicated for 40 min. Finally, samples were centrifuged for 10 min at 20,000× *g*.

### 2.8. SDS Page and Signal Quantification

Samples were loaded in a 20 cm long and 1 mm thick 17% acrylamide/bisacrylamide gel. Electrophoresis was carried out at 100 V for 16 h. The gel was fixed with a solution of acetic acid and methanol (acetic acid 10%, methanol 45%, water 45%), dyed with Coomassie blue (acetic acid 10%, methanol 45%, water 44.9%, 0.1% Coomassie blue) and dried. The labeled bands were visualized by autoradiography exposing a film for 5 days. Quantification was performed by measuring the density value of mitochondrial protein content and correcting it with the total protein load.

### 2.9. Functional Mitochondrial Characterization

In order to assess mitochondrial performance, fibroblasts were exposed for 24 h to 10 mM galactose media, where cells are forced to rely on oxidative phosphorylation for ATP production. Fibroblasts were harvested with 2.5% trypsin, (Gibco, Life Technologies™) and centrifuged at 500× *g* for 8 min. Experiments were performed in parallel with P1 and controls fibroblasts at the same passage.

### 2.10. Mitochondrial Oxygen Consumption

Oxygen consumption rates (OCRs) were measured with Agilent Seahorse XF24 Analyzer Mitostress Test (Seahorse Bioscience, Agilent, Santa Clara, CA, USA), according to manufacturer’s protocol. Briefly, 30,000–35,000 fibroblasts/well were seeded in quadruplicate in customized 24-well Seahorse cell culture plates and incubated overnight in 250 µL of 10 mM galactose medium. Growth medium was then removed, and wells were washed once with Seahorse XF Base Medium (Seahorse Bioscience) containing 10 mM Galactose, 1 mM Sodium Pyruvate and 1 mM Glutamine. Plates were incubated in this media for 30 min at 37 °C without CO_2_. The bioenergetic profile was measured obtaining the OCRs under basal condition and after the addition of oligomycin, carbonyl cyanide-4-(trifluoromethoxy) phenylhydrazone (FCCP) and rotenone-antimycin (all reagents from Sigma-Aldrich). OCR values were normalized to total cell protein content and reported as pmol/min·µg protein.

Bioenergetic Health Index (BHI) was calculated to assess the mitochondrial profile of the subjects studied by the equation described by Chacko et al. [22].

### 2.11. Cell Growth

Cell growth rate was determined through cell counting with a Neubauer chamber at the times of seeding and harvesting the cells in galactose media at 4, 7 and 10 days of growth. Results are reported as fold-change of growth from day one to ten of the experiment.

### 2.12. Mitochondrial DNA Deletion Study

Total DNA from fibroblasts was extracted using the standard phenol–chloroform extraction procedure. The assessment of mtDNA deletions was performed by long-range PCR using Phusion High-Fidelity PCR Master Mix with GC Buffer (F-532L, ThermoFisher Scientific, Waltham, MA, USA) and the following primers: forward 5′-TTAGCAAGGGAACTACTCCCA-3′ and reverse 5′-CGGATACAGTTCACTTTAGCTACCCCCAAGTG-3′. The PCR products were electrophoresed in a 0.8% agarose gel, stained with SYBR safe to analyze mitochondrial DNA integrity, and run in parallel with positive and negative quality controls.

### 2.13. Statistical Analysis

Results were expressed as mean ± standard error mean (SEM). Statistical analysis was performed through using the non-parametric Kruskal-Wallis test and the Mann–Whitney U test, with SPSS version 22 (IBM, Armonk, NY, USA). Significance was set at *p* < 0.05.

## 3. Results

### 3.1. Clinical Data of Case Reports

Two brothers were born after a term pregnancy to unrelated healthy parents. From infancy, they presented easy fatigability. P1 had permanent basal hyperlacticaemia (68–80 mg/dL; normal 5–22), leukopenia, anemia and signs of pancreatic insufficiency. Bone marrow examination revealed vacuolization of early and late erythroid and myeloid cell precursors, with more than 15% ringed sideroblasts. He was transfusion dependent for four years. Neurological examination revealed a mild but progressive proximal muscle weakness, and electromyography (EMG) disclosed a generalized myopathic pattern. P1 was clinically diagnosed with Pearson’s syndrome at 23 years old, started treatment with L-carnitine and CoQ_10_ and is currently stable at 48 years old. P2 disclosed very similar findings, including ringed sideroblasts on bone marrow examination although P2 never required transfusions. Pancreatic insufficiency, manifested as steatorrheic feces, was also confirmed and consequently, he was diagnosed with Pearson’s syndrome at 23 years old. Later, P2 developed a dysembryo-plastic neuroepithelial tumor (DNET) in the brain not requiring specific treatment since the only clinical manifestation was a mild seizure and the tumor size did not increase over time. He died at 49 years old due to respiratory failure and refractory acidosis. Previous evaluation of P1, P2 and their mother (P3) revealed multiple mtDNA deletions and MRC deficiencies in both siblings [18].

### 3.2. Genetic and Molecular Findings Revealed Two Potentially Pathogenic Variants in YARS2 Gene

Based on the previous results reported for multiple mtDNA deletions in muscle [18], P2 was studied genetically with an mtDNA maintenance gene custom panel. No pathogenic variants were found in muscle DNA from P2 in the 17 genes studied with the custom panel, that includes the majority of genes associated with pathogenicity involved in mtDNA depletion and multiple deletion syndromes. Whole mtDNA was sequenced by NGS in muscle DNA from patient P2 and no pathogenic variants were found. In addition, mtDNA integrity was analyzed in DNA from fibroblasts form patient P1, and non-multiple or single deletions were found. However, whole exome sequencing was performed in P2 DNA sample, and two potentially pathogenic heterozygous variants in *YARS2* gene were found, a single nucleotide deletion in exon 1, c.314delG (p.(Gly105Alafs*4)), which creates a premature stop codon in the amino acid 109, and a single nucleotide change in exon 5 c.1391T>C (p.(Ile464Thr)), that causes a missense variant (NM_001040436 and NP_001035526).

Sanger sequencing confirmed both variants in P2, and evidenced the same variants in P1 DNA sample. In mother’s DNA sample only the variant c.1391T>C (p.(Ile464Thr)) was found, indicating the maternal inheritance of the missense variant in both P1 and P2 brothers.

**Figure 1:**
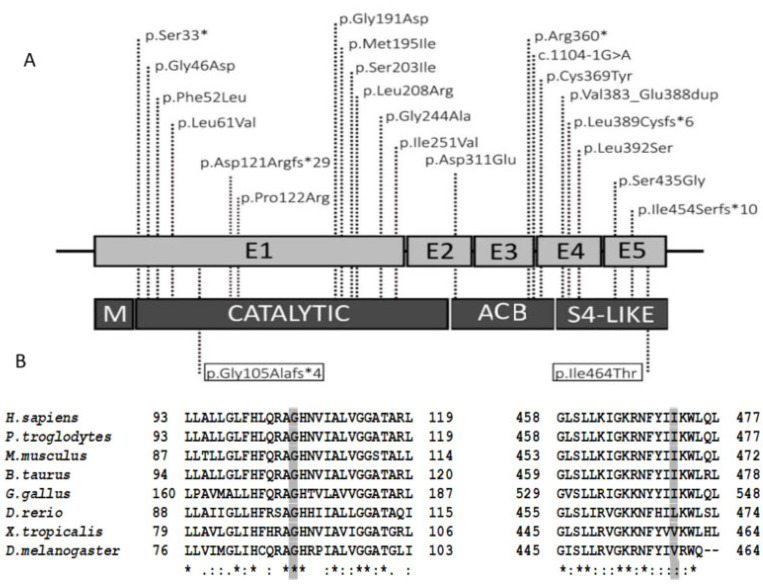
*YARS2* reported variants and evolutionary conservation. (**A**) Summary of the known *YARS2* pathogenic variants and their distribution. The two new variants are boxed within highlighted squares. E (exon), M (mitochondrial target sequence), CATALYTIC (catalytic domain), ACB (anticodon binding domain), region S4-LIKE, (ribosomal protein S4-like protein). (**B**) Amino acid conservation study. CLUSTAL online software amino acid alignment of the YARS2 regions containing the new variants. The patient’s mutated residues are shadowed, and the conserved residues are indicated with an asterisk (*).

The c.314delG variant is a frame-shift variant affecting *YARS2* gene, which is a known mechanism of disease, associated with myopathy, lactic acidosis, and sideroblastic anemia 2. This variant has been reported in population database as gnomAD Exomes with a frequency of 0.000004, less than the 0.0001 threshold for recessive gene *YARS2*. This variant is not found in gnomAD Genomes database nor in databases associated with pathology (ClinVar, HGMD). This variant is located in the catalytic domain and the amino acid conservation study indicates that glycine at the position 105 in YARS2 protein is a conserved amino acid (Figure 1). According to ACMG guidelines [23,24] this variant is classified as pathogenic (PVS1, PM2, PP3). The c.1391T>C variant predicts the change of isoleucine to threonine in the amino acid 464 of the protein. This variant has not been reported in population database as gnomAD Genomes, gnomAD Exomes database nor in databases associated to pathology (ClinVar, HGMD). In silico pathogenicity predictors classify this variant as deleterious (DANN, FATHMM-MKL, MutationAssessor, MutationTaster, PrimateAI and SIFT). This variant is located in the S4-like region and the isoleucine residue located at the site of the missense variant is partially conserved in mammals and birds (Figure 1). According to ACMG guidelines, this variant is classified as “likely pathogenic” (PM2, PM3, PP2, PP3) [23,24].

### 3.3. YARS2 mRNA Expression in Fibroblasts from Patient P1 Were Consistent with Our Genetic Findings

**Figure 2:**
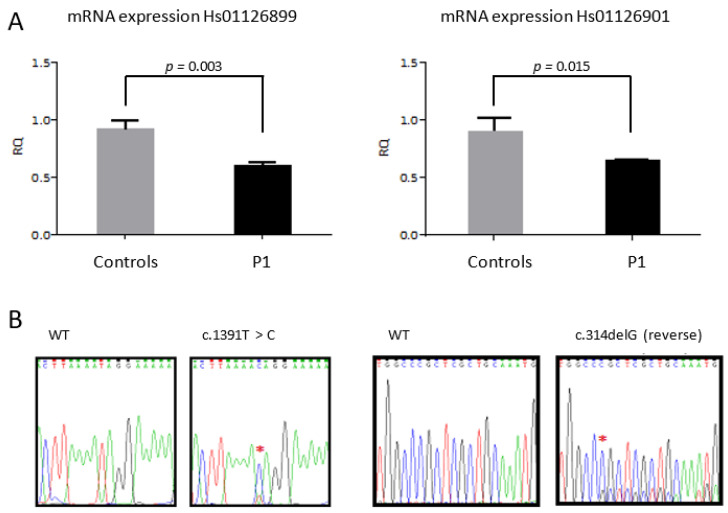
Expression levels of YARS2 transcript in fibroblasts from patient P1. (**A**) *YARS2* mRNA content of fibroblasts from P1 and 5 controls (C1-C5) using Taqman assays (located in exon 1-2 boundary (Hs01126899) and in exon 3-4 boundary (Hs01126901)). (**B**) Electropherogram of P1 cDNA sample and WT sample of c.314delG and c.1391T > C sequences.

In order to investigate if the two candidate *YARS2* variants can affect *YARS2* mRNA expression, TaqMan assays located in exon 1-2 and 3-4 boundaries of *YARS2* gene were performed in cDNA obtained from total RNA of P1. We observed a reduction of 30% in P1 *YARS2* mRNA content comparing with the mean levels of five controls (Figure 2A) with both TaqMan assays (*p* = 0.003 and *p* = 0.015 by using Hs01126899 and Hs01126901, respectively). Moreover, we analyzed by Sanger sequencing the presence of the variants in the same cDNA, electropherogram of P1 cDNA sample showed a low-height peaks started coincident with the location of the single nucleotide deletion pathogenic variant (c.314delG) (Figure 2B) while the other candidate causative variant c.1391T>C showed a high-height peak and wild type variant was difficult to distinguish from background noise, suggesting the presence of nonsense-mediated mRNA decay (NMD) that magnifying the proportion of the missense allele vs. the truncated one. Accordingly, we observed a partial reduction in the expression of *YARS2* in fibroblasts of P1. This observation indicates that the c.314delG frameshift variant should have been eliminated by nonsense mediated decay, and the 70% remaining levels correspond to the expression of the missense allele (probably a little overexpressed due to the absence of the other allelic counterpart). Moreover, these results confirm that the two variants are located in trans.

### 3.4. Decreased Mitochondrial Translation in Patient P1

**Figure 3:**
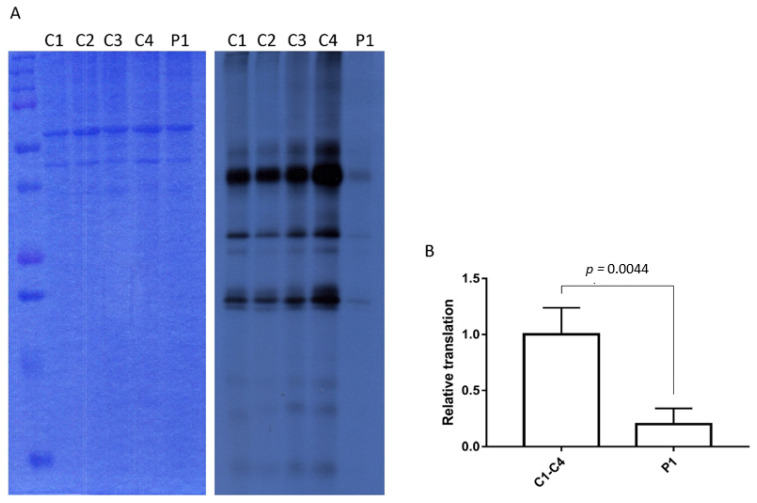
Mitochondrial translation assay. (**A**) Mitochondrial translation assay showing decreased quantity of the proteins synthesized by mitochondrial ribosomes in patient 1 (P1) compared to controls (C1-C4). Left: Coomassie gel showing the total amount of protein. Right: Exposed autoradiography film showing the mitochondrial bands. (**B**) Quantification of mitochondrial translation. Mitochondrial translation assays were performed in three independent studies in 4 control fibroblast cell lines and in the patient’s fibroblasts. The results show the quantification of mitochondrial translation of the patient fibroblasts with respect to the mean of the control fibroblasts (*n* = 4).

To assess YARS2 functionality we performed a mitochondrial translation assay in fibroblasts. We observed an 80% of reduction in the total content of mitochondrial protein translation in fibroblasts from P1, compared with control fibroblasts (Figure 3).

### 3.5. Functional Mitochondrial Characterization Revealed Associated Deficiencies in Mitochondrial Function in Patient P1

**Figure 4:**
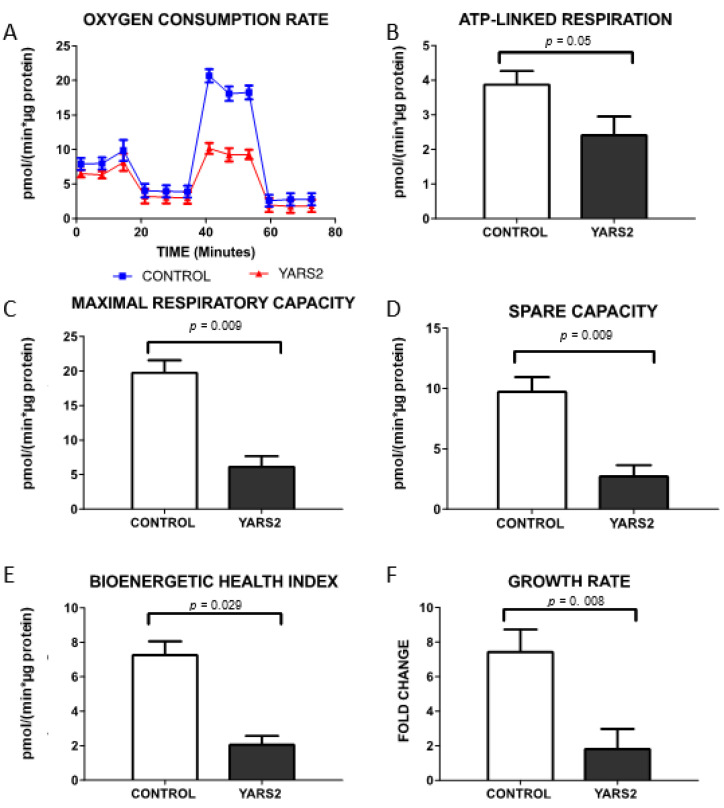
Mitochondrial functional characterization, overall cell health and growth rates. (**A**) Oxygen consumption rate was slightly decreased in fibroblast from patient P1. (**B**) The alteration is made more evident after MRC CV inhibition by oligomycin (ATP-linked respiration). (**C**) Decrease in posterior mitochondrial uncoupling (maximal respiration capacity) and (**D**) spare capacity. (**E**) BHI is significantly reduced and (**F**) growth rate in galactose media was significantly affected in the patient P1 with *YARS2* candidate variants.

To assess if the reduction in mitochondrial protein synthesis was further translated into deficiencies in mitochondrial function, we measured mitochondrial oxygen consumption in fibroblasts from P1 and in fibroblasts from 2 controls. Mitochondrial respiration rate of fibroblasts from P1 demonstrated a deficient OXPHOS activity, evidenced by decreased ATP-linked respiration (C 3.9090 ± 0.35 vs. P1: 2.4467 ± 0.5 pmol/min*µg protein; *p* = 0.05), maximal respiratory levels (C 19.93 ± 1.61 vs. P1: 6.3217 ± 1.36 pmol/min*µg protein; *p* = 0.009) and spare capacity (C 9.834 ± 1.109 vs. P1: 2.8159 ± 0.82 pmol/min*µg protein; *p* = 0.009), accompanied by a significantly decreased BHI (C 7.3350 ± 0.72 vs. P1: 2.1250 ± 0.44 pmol/min*µg protein; *p* = 0.029) (Figure 4) confirming deficiencies in mitochondrial function in patient P1.

### 3.6. Cell Growth Was Significantly Reduced in Fibroblasts from Patient P1

To further assess the effect of this *YARS2* causative variants in overall cell health performance, cell growth was measured in conditions where cell growth depends on OXPHOS function. As shown in Figure 4F, cell growth was significantly decreased in fibroblasts from patient P1 in galactose media compared with controls (Fold-Change C: 7.5 ± 1.23 vs. P1: 1.880 ± 1.1, *p* = 0.008) indicating that the variants in *YARS2* gene found in patient P1 are associated with both mitochondrial dysfunction and decreased cell growth.

## 4. Discussion

Pearson’s syndrome presents with infantile sideroblastic anemia and exocrine pancreatic insufficiency, associated to a large-scale single mtDNA deletion [24,25]. Sideroblastic anemias are rare errors of metabolism caused by mutations of genes involved in heme synthesis, alterations in iron–sulfur cluster biogenesis and transportation, defects in MRC protein synthesis and, consequently, energy production. To date, six genes have been discovered that may cause sideroblastic anemia: *PUS1*, *YARS2*, *LARS2*, *TNRNT1*, *NDUFB11* and *MT-ATP6*. Moreover, pathogenic variants in *PUS1*, *YARS2* and *MT-ATP6* were associated with MLASA syndrome, and consistently *PUS1* and *YARS2* are responsible for an almost identical clinical syndrome MLASA by altering the MRC protein synthesis by affecting distinct molecular targets [26]. *YARS2* encodes the mitochondrial tyrosyl-tRNA synthetase protein, and pathogenic variants in this gene have been associated with MLASA2 with a variable phenotype presentation. The siblings that are presented in this work were initially diagnosed with Pearson’s syndrome; however, mtDNA study did not evidence a single large-scale deletion, though multiple deletions were observed [18]. For this reason, a panel of genes related to the mtDNA maintenance was analyzed in P2 but no pathogenic variants were found. Previously to the WES approach, whole mtDNA NGS sequencing was performed in P2 to discard any pathogenic variants in mtDNA as variants in *MT-ATP6* that have been associated with MLASA.

Patient 2 (P2) WES study evidenced two new compound heterozygous variants in *YARS2* gene, c.314delG which creates a premature stop codon in the amino acid 109, and a single nucleotide change in the exon 5 c.1391T>C which predicts the substitution of isoleucine to threonine at position 464 of YARS2 protein. These variants are located in the catalytic domain and in the S4-like region of YARS2 protein. As shown in Figure 1, pathogenic variants in *YARS2* have been found distributed along YARS2 protein, including catalytic domain and S4-like regions. Missense, nonsense, frameshift and splicing variants have been described as pathogenic variants in *YARS2* gene [8,11,12,13,14,15,16,17]. Here we investigate the molecular effects of these two novel *YARS2* variants in fibroblasts of the surviving brother (P1) and found a marked decrease of *YARS2* mRNA levels attributed to nonsense-mediated mRNA decay (NMD) produced by the c.314delG *YARS2* variant. Mitochondrial protein translation was clearly reduced as OXPHOS function in P1 fibroblasts further translated into deficient overall cell health and growth and these results cannot be attributed to mtDNA deletion in P1 fibroblasts. Consequently, both variants can be classified as “pathogenic” following ACMG guidelines (achieving PS3 rule). These variants were responsible for a phenotype characterized by late-onset MLASA, herein accompanied by pancreatic insufficiency, which defines Pearson’s syndrome [24]. However, Pearson’s syndrome has classically not been associated to any point mutation and has been exclusively associated with single large scale deletion in mtDNA of unknown origin; it has also been widely described as an infant-onset and severe disease progressing to early death or to the development of Kearns-Sayre syndrome [27,28,29]. However, Gustafson et al. have recently described and suggested that the variant p.(Glu27Lys) in *SSBP1* gene can interfere with mtDNA replication and precipitate the introduction of large scale mtDNA deletions with clinical manifestations across the clinical spectrum of Pearson, Kearns-Sayre and Leigh syndromes [27].

The most common ethnicity where *YARS2* pathogenic variants are found is in Caucasian Americans, followed by Lebanese population, where four subjects have been reported [13]. A Scottish founder mutation has been recently reported, with four other cases [12]. Interestingly, before that herein reported, only one patient was from Spanish ethnicity.

Remarkably, most patients with MLASA2 syndrome have an early presentation of disease, and only three cases of adult onset and one asymptomatic carrier of *YARS2* mutation have been reported to date, such as those herein included. From the clinical point of view, there were only two cases in the literature describing intermittent diarrhea, but not other data suggesting pancreatic insufficiency has been described before the siblings herein reported [12,13]. Lactic acidosis was present in 15 cases and myopathy in 15, without coexisting in all cases from a total of 17 patients. While sideroblastic anemia may be the symptom that points toward the diagnosis in MLASA syndrome, it is not present in all patients, and the severity widely varies among subjects. Moreover, in some cases it may disappear spontaneously or fluctuate between transfusion dependent crisis and stable periods, apparently without correlating with the rate of patient survival.

As for the 55 previous reports of patients with Pearson’s syndrome, anemia was present in most cases, decreasing in case of survival, when multisystem involvement became prominent [28]. Therefore, the siblings described in this paper, harboring *YARS2* variants, present a phenotype that was clinically diagnosed as Pearson’s syndrome with an unusual benign course that, with the advent of NGS, has been finally diagnosed as MLASA2. Consequently, the concomitant presence of pancreatic dysfunction will need deeper investigation in these syndromes and may reveal a common phenotype that has been ignored to date. Our results, along the same line as others, suggest that MLASA may be only one of the clinical presentations of *YARS2* pathogenic variants, and that some other syndromic causes of unknown origin or with incompletely described molecular pathogenic routes of sideroblastic anemia could also be associated to pathogenic variants in this gene [12]. Understanding the molecular mechanisms underlying the heterogeneity of these disorders is of great importance as a first step towards developing more effective diagnostic algorithms and to discover new therapeutic targets to cure or prevent the progression of the disease.

## 5. Conclusions

As far as we know, pancreatic dysfunction has not been previously related to pathogenic variants in *YARS2* gene. The newly identified missense variant c.1391T>C, p.(lle464Thr) and the premature stop codon c.314delG (p.(Gly105Alafs*4)) variant in *YARS2* gene are responsible for a distinct phenotype of mitochondrial disease associating MLASA and pancreatic insufficiency. Therefore, the present findings open the doors to evaluate YARS2 deficits in heterogeneous forms of disease in association to isolated sideroblastic anemia, atypical presentations of MLASA [13] and even in other mitochondrial myopathies of unknown origin.

## Data Availability

Data sharing not applicable to this article as no datasets were generated or analysed during the current study.
